# Power of a dual‐use SNP panel for pedigree reconstruction and population assignment

**DOI:** 10.1002/ece3.6645

**Published:** 2020-08-10

**Authors:** Samuel A. May, Garrett J. McKinney, Ray Hilborn, Lorenz Hauser, Kerry A. Naish

**Affiliations:** ^1^ School of Aquatic and Fishery Sciences University of Washington Seattle WA USA; ^2^ NRC Research Associateship Program Northwest Fisheries Science Center National Marine Fisheries Service National Oceanic and Atmospheric Administration Seattle WA USA

**Keywords:** amplicon panel, GTseq, parentage, population assignment, Sockeye

## Abstract

The use of high‐throughput, low‐density sequencing approaches has dramatically increased in recent years in studies of eco‐evolutionary processes in wild populations and domestication in commercial aquaculture. Most of these studies focus on identifying panels of SNP loci for a single downstream application, whereas there have been few studies examining the trade‐offs for selecting panels of markers for use in multiple applications. Here, we detail the use of a bioinformatic workflow for the development of a dual‐purpose SNP panel for parentage and population assignment, which included identifying putative SNP loci, filtering for the most informative loci for the two tasks, designing effective multiplex PCR primers, optimizing the SNP panel for performance, and performing quality control steps for downstream applications. We applied this workflow to two adjacent Alaskan Sockeye Salmon populations and identified a GTseq panel of 142 SNP loci for parentage and 35 SNP loci for population assignment. Only 50–75 panel loci were necessary for >95% accurate parentage, whereas population assignment success, with all 172 panel loci, ranged from 93.9% to 96.2%. Finally, we discuss the trade‐offs and complexities of the decision‐making process that drives SNP panel development, optimization, and testing.

## Graphical abstract

The use of high‐throughput, low‐density sequencing approaches has dramatically increased in recent years in studies of eco‐evolutionary processes in wild populations and domestication in commercial aquaculture. Here, we detail the use of a bioinformatic workflow for the development of a dual‐purpose SNP panel for parentage and population assignment in Alaskan Sockeye Salmon. We discuss the trade‐offs and complexities of the decision‐making process that drives SNP panel development, optimization, and testing.

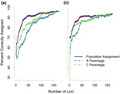

## 1 INTRODUCTION

Single nucleotide polymorphisms (SNPs) have become commonplace in ecological and evolutionary studies and are particularly useful for pedigree reconstruction and individual assignment to populations (Seeb, Carvalho, et al., 2011). The use of pedigrees in evolutionary studies has rapidly expanded, especially in investigations related to fitness and genetic diversity of wild systems. For example, pedigrees may be used to estimate inbreeding depression, relatedness, and individual reproductive success, and they are used in parentage‐based population assignment (Bradshaw, 2017; Pemberton, 2008; Richards‐Zawacki, Wang, & Summers, 2012) and close‐kin mark–recapture studies (Bravington, Grewe, & Davies, 2016). While useful, the reconstruction of wild pedigrees can be constrained by processing time and the expense of obtaining molecular data for thousands of individuals. There may also be limitations associated with sampling all individuals in a population over multiple generations because of logistic constraints. Individuals might disperse between interconnected populations, which can confound data analyses but may also provide key information on the contribution of immigrants to population fitness (Peterson, Hilborn, & Hauser, 2014). As such, coupling pedigree reconstruction with approaches that assign individuals to their population of origin (population assignment) can aid in describing broader evolutionary processes across interconnected populations.

Despite the benefits of combining pedigrees with population assignments, the two objectives require different population allele frequencies for optimal performance. Pedigree reconstruction benefits from loci with high minor allele frequencies (Anderson & Garza, 2005; Holman, Garcia de la serrana, Onoufriou, Hillestad, & Johnston, 2017), whereas population assignment benefits from highly differentiated loci between populations (Anderson, 2010). This ascertainment bias (Bradbury et al., 2011; Seeb, Templin, et al., 2011), introduced by selecting loci for a specific purpose, may limit their applications in other analyses and produce important trade‐offs in the design of multi‐use SNP panels.

Several high‐throughput sequencing technologies have been developed in recent years that allow thousands of individuals to be pooled in a single lane of sequencing and genotyped at hundreds to thousands of SNP loci (e.g., GTseq, Campbell, Harmon, & Narum, 2015; Rapture, Ali et al., 2015; and MTAseq, Onda, Takahagi, Shimizu, Inoue, & Mochida, 2018). These approaches require the selection of specific subsets of loci and optimization of primers prior to sequencing but substantially reduce the cost of genotyping per individual, because the number of loci that can be reasonably included is limited to a few hundred (Meek & Larson, 2019) by problems arising from nonspecific heterodimer formation (Aykanat, Lindqvist, Pritchard, & Primmer, 2016), unequal amplification (McKinney, Pascal, et al., 2020), and minimum required coverage.

Several studies have outlined best practices for the selection and optimization of marker panels (Holman et al., 2017; Liu, Palti, Gao, & Rexroad, 2016; McKinney, Pascal, et al., 2020), including for multiple downstream applications (Aykanat et al., 2016). The effect of different locus numbers and population sizes on parentage (Harney et al., 2018; Holman et al., 2017) and population assignment (Baetscher, Clemento, Ng, Anderson, & Garza, 2018; McKinney, Seeb, & Seeb, 2017) is also well known. However, panels are usually either assembled for a single purpose or as single‐purpose, bioinformatically separated modules in combined panels (Aykanat et al., 2016). To our knowledge, the combined power of entire multi‐purpose panels for single objectives has not been tested.

Single nucleotide polymorphisms panel‐based approaches have been increasingly used in salmonids (Holman et al., 2017; Janowitz‐Koch et al., 2019; Liu et al., 2016; McKinney, McPhee, Pascal, Seeb, & Seeb, 2020; Steele et al., 2017), a group of fishes that has been the subject of evolutionary ecology studies in natural systems, not only because of their commercial, ecological and cultural significance, but also because well‐defined spawning populations in freshwater habitats and relatively easy sampling greatly facilitate such studies. Sockeye Salmon (*Oncorynchus nerka*) in particular have been the focus of many behavioral, ecological, and evolutionary studies that use both population assignment and pedigree analyses (i.e. Lin, Hard, Hilborn, & Hauser, 2017; Lin et al., 2016; Peterson et al., 2014). So far, these studies have relied primarily on microsatellite markers in small populations over one or two generations. However, extended eco‐evolutionary questions over many generations necessitate the efficient genotyping of thousands of individuals at loci capable of both accurate pedigree reconstruction and individual assignment to population of origin. If the design of such panels is sufficiently successful and genotyping costs are sufficiently low, similar approaches may be possible for species where large‐scale pedigrees and population assignment were hitherto deemed impossible; for example, rockfishes (*Sebastes* sp., Baetscher et al., 2019) or tuna (T*hunnus maccoyii*, Bravington et al., 2016).

Here, we describe the development of a GTseq amplicon primer panel for rapid and effective genotyping of Sockeye Salmon to address two distinct objectives: pedigree reconstruction and determination of population of origin. We demonstrate the use of a streamlined workflow (McKinney, Pascal, et al., 2020) and detail the processes of marker selection, optimization, and testing. Furthermore, we investigate the effects of ascertainment bias on downstream applications, in an effort to determine whether dual‐use SNP panels benefit from the combination of markers or effectively represent independent sets of loci that do not add much power to the alternative application.

## 2 METHODS

### 2.1 Study system

Tissue samples were collected from Sockeye Salmon spawning in two small creeks (A and C Creeks; 250 and 350 m long, respectively), approximately 1 km apart on Little Togiak Lake, Alaska (Peterson et al., 2014). We utilized dorsal fin tissue samples from 2010 for panel development and 2009 for panel optimization and power verification. Tissue samples were collected from nearly every fish returning to the creeks throughout the spawning season and stored in 100% ethanol (Peterson et al., 2014). *F*_ST_ values, estimated by Lin, Quinn, Hilborn, and Hauser (2008) using microsatellite markers, range from 0.02 to 0.04 (Lin et al., 2008).

### 2.2 Ascertainment of reference sequences for panel development: RAD sequencing and genotyping

**FIGURE 1:**
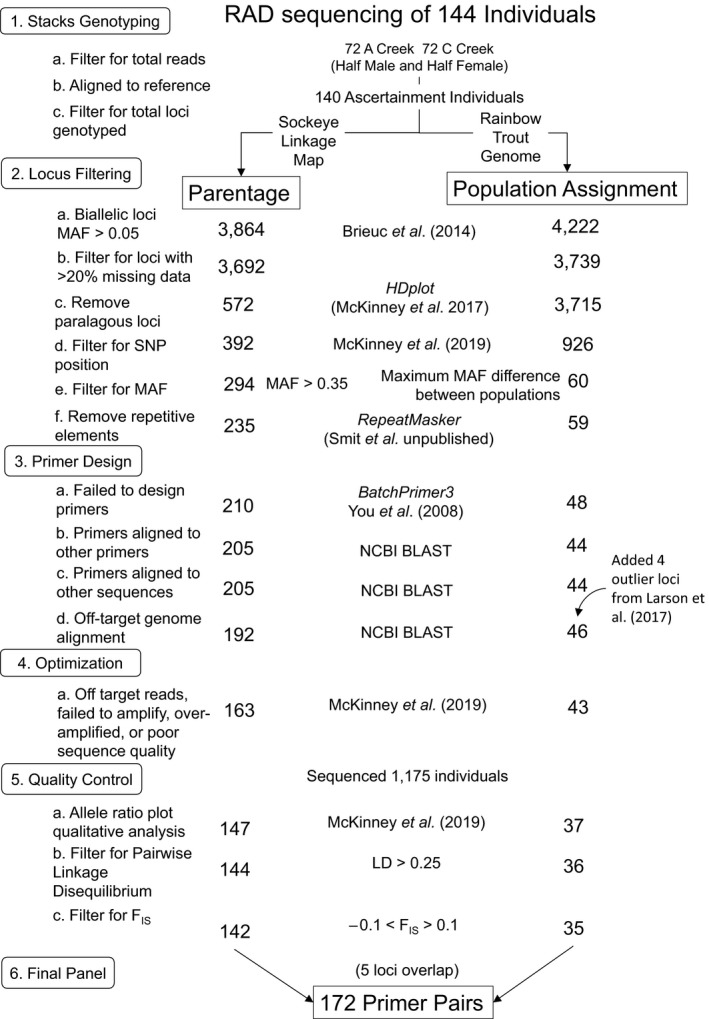
Schematic of the workflow and the results for SNP discovery, filtering, primer design, optimization, and quality control for a 172 SNP GTseq primer panel for use in individual assignment to population of origin (population assignment) and assignment of parent–offspring pairs (parentage). Numbers give the number of loci in the panel after each filtering step for use in parentage (left) or population assignment (right). Methods used for each step are given in the middle column

DNA was extracted from 144 individuals representing both A and C creeks (Figure 1 Step 1) using Qiagen DNeasy Blood and Tissue kits (Qiagen). Two RAD‐seq libraries (Baird et al., 2008) were prepared following the bestRAD protocol: the first step of the RAPTURE protocol (Ali et al., 2015) using the *Sbf1* restriction enzyme. Paired‐end 2 × 100‐base pair sequencing was performed on an Illumina HiSeq4000. Raw RAD sequencing data were processed using *Stacks* (v.1.42, Catchen, Hohenlohe, Bassham, Amores, & Cresko, 2013) following the bioinformatics pipeline described in Waters et al. (2018). Briefly, forward reads were demultiplexed and trimmed to 95bp using *process_radtags*. Individuals with fewer than 200,000 total reads were excluded from further analysis (Figure 1, step 1a). No assembled genome is yet available for Sockeye Salmon. Instead, reads were aligned to both the Rainbow Trout reference genome (*O. mykiss*, GenBank assembly Accession GCA_002163495) and a Sockeye Salmon linkage map (Larson et al., 2015, based on haploid data) using *Bowtie*2 (v.2.3.3.1, Langmead & Salzberg, 2012), allowing up to three base pair mismatches. These two alignments were treated as separate pipelines during the remaining panel development steps (Figure 1). Loci for each individual were identified using *pstacks* with the bounded‐error SNP calling model, a minimum read depth of 10, and the default error rates. Individuals with <19,000 loci in the *O. mykiss* pipeline or 4,500 loci in the *O. nerka* linkage map pipeline were excluded from further analysis, representing breaks in the distribution of loci (Figure 1, step 1c). The 10 individuals from each population with the highest read depth and coverage were used to construct a catalog of loci in *cstacks*; this subset was used to reduce the risk of including false polymorphisms in the catalog (as in Waters et al., 2018). Loci from individuals aligned to either the *O. mykiss* genome or Sockeye linkage map were matched to their respective catalogs using *sstacks*. Finally, genotypes were assigned using *populations*, with a minimum read depth of 10.

### 2.3 Locus filtering and SNP selection

Following *Stacks* genotyping, all biallelic loci were regenotyped using a custom Python script to verify and correct genotypes, therefore minimizing potential bias in maximum likelihood genotype calls in *Stacks* due to differences in read depth between two alleles at a locus (Brieuc, Waters, Seeb, & Naish, 2014, but also Waters et al., 2018). Loci were excluded if they had a minor allele frequency of <0.05 in either creek population (Figure 1, step 2a). Loci genotyped in ≤80% of individuals, and individuals with ≥50% missing data were excluded from further analyses (Figure 1, step 2b).

We followed the filtering and SNP selection methods in McKinney, Pascal, et al. (2020) to reduce the effects of unequal locus amplification, cross‐amplification of primer pairs, and off‐target amplification (Figure 1, step 2). Loci were filtered using *HDplot* (McKinney, Waples, Seeb, & Seeb, 2017); https://github.com/gjmckinney/HDplot ) to ensure no undifferentiated paralogous loci were included in the panel. To permit sufficient sequence length for primer development, polymorphic SNP loci were further filtered to exclude loci within 15 base pairs of the start or end of the 95bp RAD sequence. The program *RepeatMasker* (Smit et al. unpublished v.4.0.6) was used to identify and remove loci in low complexity regions or transposable elements, using the default parameters. To reduce risk of off‐target amplification, loci aligned to the *O. mykiss* genome were excluded if they matched to more than 6 unique regions. This represented a break in the distribution of matches per locus and was used as a qualitative threshold.

Loci were included in the panel to serve two primary purposes: pedigree reconstruction and population assignment. SNPs for parentage were selected from loci identified through alignment to a Sockeye Salmon linkage map (Larson et al., 2015), as no genome has yet been assembled for Sockeye. Loci were excluded if they had minor allele frequencies <0.35 in either population, as an allele frequency close to 0.5 maximizes the power for parentage (Anderson & Garza, 2005). Conversely, the power to assign population of origin increases when loci are highly differentiated between populations (Anderson, 2010). We anticipated that alignment to the Sockeye Salmon linkage map may have caused population‐specific ascertainment bias, as the linkage map comprised loci that were polymorphic in few families and from different populations than the present study, resulting in little differentiation between our study populations (Larson et al., 2015). Therefore, discovery of SNPs for population assignment was instead derived from loci aligned to the *O. mykiss* genome, and SNPs that had the greatest difference in minor allele frequency between the two creek populations were selected. Beach and creek populations of Sockeye Salmon represent different morphotypes, but they often intermingle close to the spawning streams (Lin et al., 2008; Peterson et al., 2014). Therefore, an additional set of outlier loci differentiating creek and beach populations (Larson et al., 2017) were included to differentiate these highly divergent populations. These outlier loci were included for use in future studies, but their utility in differentiating beach and creek populations is not addressed further here.

### 2.4 Primer design

Primers for all putative loci were designed in *BatchPrimer3* (v.1.0, You et al., 2008), with no optimum fragment or primer size. We followed the protocol of McKinney, Pascal, et al. (2020) to optimize primers prior to sequencing. NCBI's BLAST (Altschul et al., 1997) was used to align all putative primer sequences to all putative amplicon sequences, to prevent potential interactions between the two. The putative primer sequences were also aligned to the *O. mykiss* genome to identify and exclude primers that may bind to more than one region of the genome.

### 2.5 Optimization

The final panel of primers was used to construct GTseq libraries for 24 individuals, following the methods in Campbell et al. (2015). Paired‐end 2 × 75‐base pair sequencing was performed on an Illumina MiSeq. We used scripts from Campbell et al. (2015) to genotype individuals and to identify and exclude primers which overamplified loci compared to other primer pairs. These scripts use the forward primer and an in‐silico probe to count amplicon‐specific sequences for each allele and assign genotypes based on observed allele ratios. This step helped to reduce off‐target sequencing and increase read depth for the remaining loci by redistributing reads. We used custom scripts from McKinney, Pascal, et al. (2020) to identify primer interactions (Figure 1, step 4) that were not identified by BLAST (Figure 1, step 3b). In instances where different sets of primers interacted to amplify off‐target regions, all but one of the interacting primer sets were excluded. To further reduce off‐target amplification and amplification of duplicated loci, allele ratios at each locus were analyzed following the allele ratio plotting methods in McKinney, Pascal, et al. (2020); this step ensured that allele ratios conformed to those expected at single loci. In cases where loci did not fit expected allele ratios, the in‐silico bioinformatic probe in the genotyping script of Campbell et al. (2015) was extended from 15 to 30 base pairs on either side to better exclude off‐target sequence. The genotyping script was rerun, and individuals were regenotyped with these new parameters. If this in‐silico probe extension did not result in expected allele ratios, the locus was removed from the panel. The panel of primers was tested a second time with a set of 96 individuals using single‐end 100‐base pair sequencing on an Illumina MiSeq. Filtering was repeated for off‐target and overamplified loci, as above. A final set of primers was assembled.

### 2.6 Quality control

To test the efficacy of the panel on a high‐throughput dataset, DNA was extracted from 618 A Creek and 422 C Creek individuals from 2009, using Nexttec 1‐Step DNA Extraction Kits (Nexttec Biotechnologie), and GTseq libraries were prepared following the methods in Campbell et al. (2015). Single‐end 100‐base pair sequencing was performed on an Illumina HiSeq4000 and individuals were genotyped as above, using custom scripts from Campbell et al. (2015). A final round of locus filtering excluded loci from downstream analyses with unusual allele frequency ratios, following McKinney, Pascal, et al. (2020), as above. Pairwise linkage disequilibrium (*R* ^2^) was calculated between all SNP loci using the method from Hilland Robertson (1968) in the *LDcorSV* package (Desrousseaux, Sandron, Siberchicot, Cierco‐Ayrolles, & Mangin, 2013) in R (R Core Team, 2013). One locus per linked pair was excluded if pairwise *R* ^2^ values were >0.25. *F*_IS_ values were calculated at each locus for each creek population using the *Hierfstat* package (Goudet, 2005) in R, and loci were excluded if *F*_IS_ values were consistently >0.1 or <−0.1 in both populations.

### 2.7 Power analyses

Power analyses were run to test the performance of loci for each of our two objectives: to identify parent–offspring pairs and assign individuals to their population of origin. We used the training and hold‐out method of Anderson (2010) for both parentage and population assignment to minimize high‐grading bias. This method divides the dataset of empirical genotypes into two subsets: one to calculate allele frequencies for locus ranking (the training dataset) and another to calculate allele frequencies from which populations are simulated (the hold‐out dataset). We evenly divided the 1,040 sequenced individuals into hold‐out and training datasets by population, which were used in all subsequent analyses. The training dataset was used to rank loci by power for parentage and population assignment. The hold‐out dataset was used to calculate population allele frequencies for use in simulating populations. As there is an approximately 3%–12% dispersal rate between these two creek populations (Peterson et al., 2014; Peterson, Hilborn, & Hauser, 2015), individuals in the training and hold‐out datasets were cross‐referenced with dispersal data from Peterson et al. (2014) to remove immigrants. We included only philopatric individuals, which returned to the same creek where all identified parents were born.

We tested the power of different numbers of panel loci to assign parent–offspring pairs using the unknown‐sexes simulation module in the program Cervus3 (v. 3.0.7., Kalinowski, Taper, & Marshall, 2007; Marshall, Slate, Kruuk, & Pemberton, 1998) with 100 model repetitions and 8 subsets of panel loci ranked in decreasing order of minor allele frequency in the training dataset. Populations of sizes of *N* = 100 and *N* = 500 parents were simulated to generate equivalent population sizes (100 and 500) of offspring using allele frequencies in the hold‐out dataset. To simulate a realistic population, training and hold‐out datasets for this parentage analysis included only the C Creek population, assuming that 70% of parents were sampled, a mistyping rate of 0.005 (Campbell et al., 2015), and strict assignment (>95% probability). Using empirical pedigree data from Peterson et al. (2014), we estimated input parameters for the proportion of related individuals (0.0143), average relatedess among relatives (0.447), and rate of inbreeding (0.00). We estimated LOD scores of both single‐parent (parent–offspring pair) and parent‐pair (trio of two parents, one offspring) assignments. Additionally, we examined the effect of ranking panel loci on locus‐specific nonexclusion probabilities (NEP, Kalinowski et al., 2007). NEP measures the probability of not excluding a false parent at a given locus and can be used to measure locus‐specific power for parentage. We compared per‐locus first‐parent NEP ranked in order of decreasing power for parentage (decreasing minor allele frequency) with NEP in order of decreasing power for population assignment (decreasing difference in MAF between populations).

The accuracy of the population‐specific loci in assigning individuals to their population of origin (A Creek or C Creek) compared to the broader panel was tested using the R package *rubias* (Moran & Anderson, 2018). Population assignment was performed using the leave‐one‐out approach of Anderson, Waples, and Kalinowski (2008), with 500 simulated individuals based on the hold‐out dataset. Allele frequencies were calculated from 100 individuals, randomly resampled 100 times from each population in the training dataset. Loci were ranked by allele frequency for each of these 100 draws. For each unique locus ranking order, the leave‐one‐out assignment procedure was repeated 10 times. Therefore, there were 1,000 model repetitions (100 × 10) for each of 172 different panel subsets. Each subset added one additional locus to the panel, and they were used to examine the number of loci needed for accurate population assignment. Mean population assignment accuracy and 95% confidence intervals were calculated from the 1,000 repetitions of each panel subset. We then tested whether our ranking system during panel development reduced the number of panel loci necessary for accurate population assignment using the same simulation conditions. In this case, we used loci ranked in order of decreasing power for parentage in each population instead of power for population assignment.

## 3 RESULTS

### 3.1 Marker selection and optimization

**FIGURE 2:**
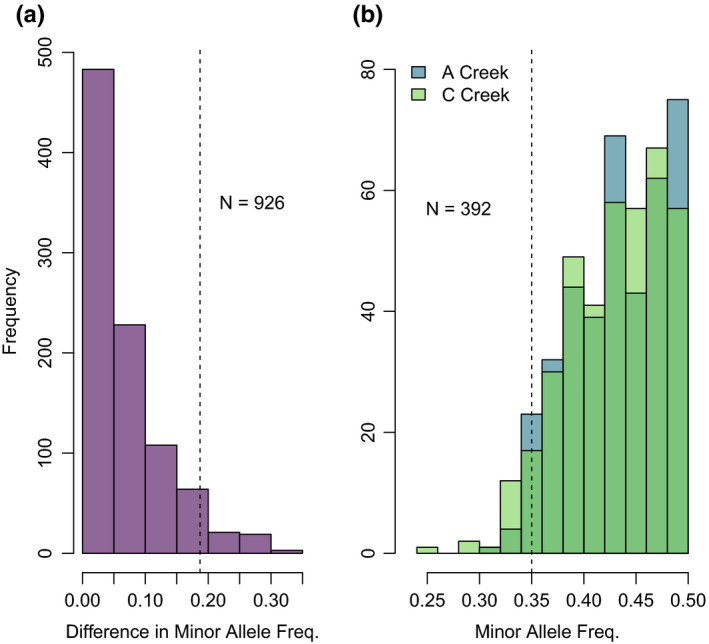
(a) Distributions of the difference in minor allele frequency between the A Creek and C Creek populations of Sockeye Salmon at 926 biallelic loci screened for use in population assignment (left), and (b) distributions of the minor allele frequencies at 392 biallelic loci screened for use in parentage (right). Loci were included following initial screening steps (step 2d in Figure 1). Loci to the right of the dashed lines were included in downstream analyses (step 2e in Figure 1)

No individuals had fewer than 200,000 reads; thus, 72 individuals from C Creek and 72 from A Creek were used for further panel development, with an average read depth of 3.8 million reads per individual. Four individuals were removed prior to genotyping in the *Stacks* module *populations* due to a small number of identified loci (Figure 1, step 1c). Alignment to the *O. mykiss* genome, followed by subsequent genotyping, yielded 4,222 biallelic RAD loci. Alignment to the *O. nerka* linkage map (Larson et al., 2015) yielded 3,864 biallelic loci. The numbers of loci remaining after each screening step are given in Figure 1. After completing the initial locus filtering steps (Figure 1, step 2, following McKinney, Waples, et al., 2017), 294 loci with minor allele frequencies >0.35 across both populations were selected for parentage, whereas 60 loci with maximum differences in allele frequencies between the two creek populations (0.19–0.30) were selected for population assignment (Figure 2).

The final panel comprised 142 loci for parentage and 35 loci for population assignment after the final quality control step (Figure 1 step 5). Five of these loci were shared between both panel subsets; therefore, the final panel comprised 172 primer pairs (Table S1). Primer lengths ranged from 16 to 26 base pairs. A total of 1,175 individuals from 2009 were sequenced with the final panel of 172 loci, and of these, 1,165 individuals (>99%) were successfully genotyped at more than 155 loci (90% of loci).

### 3.2 Panel accuracy

**FIGURE 3:**
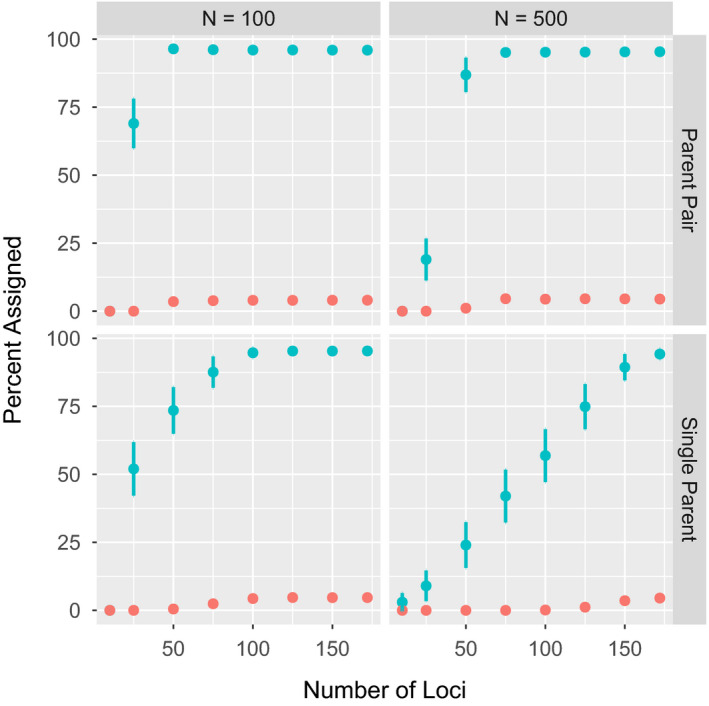
Percentage correct parentage assignment across variable numbers of SNP loci ranked from high to low minor allele frequencies in training dataset. The mean percent of offspring correctly assigned (blue) or misassigned (red) to parents (*y*‐axis) is shown for 100 model repetitions. Error bars indicate 95% confidence intervals. Proportion of parents sampled was set to 0.7, and the percentage correct assignment is given as a proportion of sampled parents. The percentage misassignment was always <5% and represents the proportion of cases where a false parent was assigned, whether the true parent was sampled or not

**FIGURE 4:**
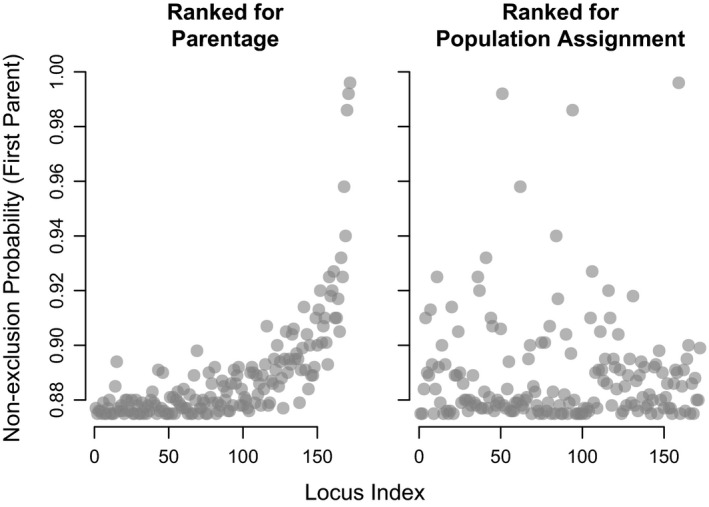
First‐parent nonexclusion probabilities (NEPs, the probability of not excluding a false parent) in parentage analyses. Comparisons between parentage (left) and population assignment (right) panels NEPs (*y*‐axis) for each ranked panel locus (*x*‐axis) were calculated in Cervus3 (Kalinowski et al., 2007). Loci are ranked according to empirical minor allele frequencies high to low in the C Creek population (parentage markers, left) and allele frequency difference between the A and C Creek populations, high to low (population assignment markers, right)

In parentage simulations, the use of the full panel of 172 loci resulted in correct assignment >95% in all cases (Figure 3). A minimum of 50 loci were needed to achieve >95% parentage accuracy in parent‐pair analyses with a population size of 100; in contrast, 75 loci were needed for a population size of 500. Assignment of offspring to parent‐pairs required fewer loci than single‐parent assignments (Figure 3) because trios (2 parents/1 offspring) are easier to identify than parent–offspring pairs. In the large population, single‐parent assignment required all 172 loci with an almost linear increase in assignment accuracy as loci were added. Misassignment (assignment to a false parent) slightly increased with number of panel loci but never exceeded 5.0%. Ranking loci by decreasing minor allele frequencies resulted in an increase in NEP (Figure 4). However, when loci were ranked by decreasing allele frequency differences between populations, NEP was randomly distributed.

**FIGURE 5:**
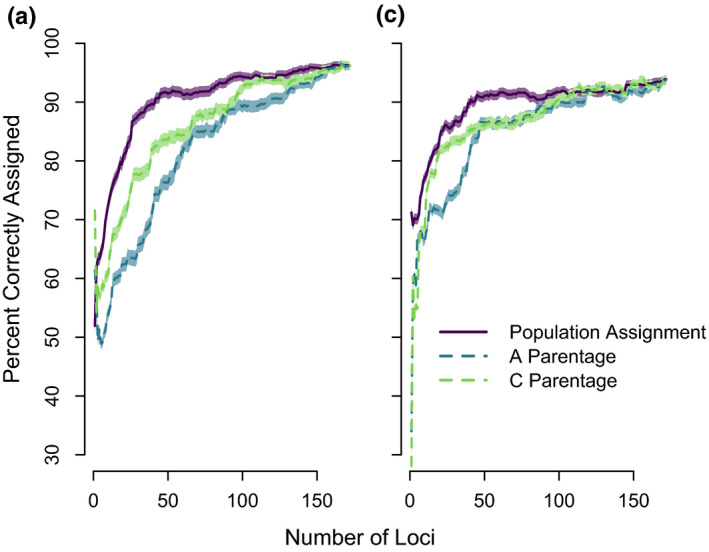
Analysis of population assignment accuracy (*y*‐axis) between marker panels developed for population assignment and parentage. A number of markers used in the analysis (*x*‐axis) are ranked by empirical allele frequency differences between populations (high to low, population assignment markers) and by within‐population allele frequency (high to low, parentage panels) for each population (A and C Creeks). Shaded regions represent 95% confidence intervals of 1,000 model repetitions of 500 simulated individuals

Population assignment success was high in both creek populations when the full panel was used (Figure 5). In A Creek, 96.2 ± 0.65% of individuals and in C Creek, 93.9 ± 0.69% of individuals were correctly assigned to their population of origin. Not all panel loci were needed to achieve high population assignment success rates, as assignment success reached an asymptote after approximately 80 loci in both creeks, and only 43 loci were necessary to achieve population assignment of >90% in both creeks. However, use of the 35 loci specifically selected for population assignment was insufficient to achieve maximum assignment accuracy. When loci were ranked by decreasing power for parentage in each of the creek populations, more loci were needed to obtain high assignment accuracy: Approximately 100 loci were needed to achieve >90% accuracy, compared to 40 when ranked by power to assign population (Figure 5). For all panel loci, mean locus‐specific *F*_ST_ was 0.016 ± 0.021 (Table S1).

## 4 DISCUSSION

Here, we developed an amplicon panel of SNP loci in Sockeye Salmon for use in pedigree reconstruction and in assignment of individuals to population of origin. These two analyses can be combined to identify both natal and immigrant individuals in a population, ultimately supporting the study of processes such as gene flow, dispersal, and inbreeding. We described the detailed application of a bioinformatics workflow (McKinney, Pascal, et al., 2020) in panel optimization and quality control in downstream applications. We identified a final panel of 142 loci for use in parentage and 35 loci for use in individual assignment to population of origin. Genotyping success rate was high (>99% of individuals were genotyped at >90% of loci), highlighting the efficacy of the panel. We found that only 50 loci were necessary to achieve >95% parent‐pair accuracy in populations of size *N* = 100, and 75 loci when *N* = 500. Population assignment success ranged from 93.9% to 96.2% in simulated populations when using all 172 panel loci.

Simulations on population sizes of up to 500 parents and 500 offspring were within the approximate range of the empirical test populations (Lin et al., 2016). The finding that population size had no effect on parent‐pair assignment accuracy after 75 loci across these populations reveals the likely range of markers needed for similar panels. Parentage assignment in larger populations requires more loci for accuracy. The findings reported here generally agree with both theoretical (Anderson & Garza, 2005) and empirical (Holman et al., 2017; Liu et al., 2016) studies that show between 50 and 100 loci are sufficient to achieve high parentage success. In a previous study on A Creek Sockeye Salmon, approximately 80 randomly selected polymorphic SNP loci were sufficient to achieve high parentage assignment success (Hauser, Baird, Hilborn, Seeb, & Seeb, 2011). In contrast, we deliberately selected loci with high MAF, which reduced the number of loci necessary for parentage to between 50 and 75. Our finding that single‐parent assignments require more loci than parent‐pairs is consistent with past findings, as larger family groups are easier to identify than single related pairs (Baruch & Weller, 2008; Hauser et al., 2011; Wang, 2007). Although we simulated related individuals based on empirical estimates, more loci may be required in smaller populations comprising a higher number of relatives. However, the linear increase of parentage assignment success across the entire range of loci suggests only a minor effect of locus ranking. Interestingly, there are an optimum number of loci because too many loci increased misassignment rate without substantially improving correct assignment. As expected, first‐parent nonexclusion probabilities (NEP) increased with decreasing minor allele frequency. However, NEP values showed no trend when loci were ranked by power for population assignment, demonstrating that the trade‐off between these two applications was not as straightforward as one might expect and that loci selected for population assignment may still be useful for parentage.

Population assignment tests revealed that as few as 40 loci obtained population assignment accuracies >90%. Analyses based on ranking revealed that locus selection based on maximum divergence was a viable approach to improve assignment success. Given low F_ST_ values between the A and C creek populations (approximately 0.02–0.04, Lin et al., 2008), more loci were needed than between more divergent populations (Helyar et al., 2011; Morin, Martien, & Taylor, 2009). Therefore, we would recommend following well‐established methods to estimate the number of putative SNPs necessary to successfully assign individuals to populations a priori, based on known population F_ST_ estimates (e.g., Sylvester et al., 2018). We broadly recommend including approximately twice the number of SNPs necessary to account for locus dropout through optimization and quality control. Lower assignment success with loci selected for the parentage panel demonstrated the effects of ascertainment bias, but also showed that such loci are still useful for the population assignment.

While our bioinformatics protocol was successful and efficient, there are several changes and trade‐offs we would recommend considering for the development of multi‐use GTseq panels. To avoid loci which may confound parentage and population assignment, we added a step to the protocol of McKinney, Pascal, et al. (2020) which excluded putative panel loci with *F*_IS_ values >0.1 or <−0.1 and were in linkage disequilibrium (LD) at the final quality control step (Figure 1, Step 5). We excluded loci in LD because they can confound parentage and population assignment analyses (Helyar et al., 2011); however, loci in LD can be informative for analyses other than parentage and population assignment—for example, they can be diagnostic for genomic features (i.e., inversions); thus, they may be considered for inclusion when developing similar panels.

We again recommend including at least twice as many loci in the primer design and optimization steps (Figure 1, Step 3) than would be needed for downstream applications, to buffer against locus drop out from many filtering steps. However, there are trade‐offs to consider between number of putative primers to include, cost of development, and panel efficacy. Primers currently cost approximately $20 per pair (Integrated DNA Technologies, Inc.). The fewer loci included in a multiplex PCR reaction, the lower the likelihood of primer–primer interactions. Therefore, it is important to evaluate the relative cost of including additional primers in a test panel versus additional locus discovery and testing. For example, we included only the 60 top ranked loci in the test population assignment panel (Figure 1 Step 2e). However, we retained only 35 of these loci, which were insufficient to achieve maximum population assignment accuracy on their own. In retrospect, the screening of at least 100–150 loci would have cost an additional $800–1,800. However, this oversight did not affect downstream applications in the final panel because the inclusion of parentage loci still resulted in >95% population assignment success. A panel developed exclusively for population assignment, would have cost approximately $4,000 in additional optimization. This redundancy may be considered an additional benefit of multi‐use SNP panels.

Although we used exclusively biallelic single‐SNP loci, we recommend evaluating the use of microhaplotypes—multiple SNPs within an amplicon whose individual alleles can be combined into haplotype alleles. These markers are multiallelic and thus substantially increase the power for relationship inference (Baetscher et al., 2018) and genetic stock identification (McKinney, Seeb, et al., 2017). Thus, microhaplotyes may also add increased power to studies where fewer amplicons are available, and because GTseq amplicons are very short (40–100 base pairs), they may be suitable for analysis of degraded DNA (Schmidt, Campbell, Govindarajulu, Larsen, & Russello, 2019). In small populations, however, this advantage may vary as genetic drift can act to reduce the number of SNPs and resulting microhaplotype alleles per locus relative to large populations. Very few loci had two or more SNPs in the small populations we screened (15 loci with two SNPs and one locus with three SNPs). In addition, genotyping multiple SNPs per locus can be challenging. That said, Baetscher et al. (2018) and McKinney, Pascal, et al. (2020) have developed bioinformatic pipelines for this task (Microhaplot and GTscore, respectively). Our panel yielded high accuracy in both parentage and population assignment analyses using only single‐SNP loci. Systems with larger population sizes, systems with less genetic variation, or different downstream applications may require additional power and may benefit from the inclusion of microhaplotypes.

In conclusion, development of multi‐purpose panels should consider trade‐offs in locus selection for different applications. However, these trade‐offs may be less severe than expected. As such, loci developed for one purpose may still add power to other applications, such that the total number of loci needed may be substantially less than the sum of loci needed for each application. Inclusion of such multi‐use loci may reduce the number of primers to test, increase the number of samples per sequencing lane, and ultimately reduce the cost of large‐scale applications. In the case that some loci may decrease power for certain applications, it is simple to bioinformatically subset the panel after sequencing (i.e., Aykanat et al., 2016; McKinney, Pascal, et al., 2020). Therefore, multi‐purpose panels benefit from inclusion of a wide range of loci. We believe the information presented here on the processes involved in SNP selection, optimization, and quality control will be of use to others in designing successful multi‐use panels for other species or populations.

## CONFLICTS OF INTEREST

The authors declare no conflicts of interest.

## AUTHOR CONTRIBUTION

**Samuel A. May:** Conceptualization (lead); Data curation (lead); Formal analysis (lead); Investigation (lead); Methodology (lead); Project administration (lead); Validation (lead); Visualization (lead); Writing‐original draft (lead); Writing‐review & editing (lead). **Garrett J. McKinney:** Conceptualization (supporting); Formal analysis (supporting); Investigation (supporting); Methodology (equal); Writing‐original draft (supporting); Writing‐review & editing (supporting). **Ray Hilborn:** Conceptualization (supporting); Data curation (equal); Funding acquisition (lead); Resources (supporting). **Lorenz Hauser:** Conceptualization (equal); Formal analysis (supporting); Funding acquisition (supporting); Investigation (supporting); Methodology (equal); Project administration (equal); Supervision (equal); Validation (supporting); Writing‐original draft (supporting); Writing‐review & editing (supporting). **Kerry A. Naish:** Conceptualization (equal); Formal analysis (supporting); Funding acquisition (supporting); Investigation (supporting); Methodology (supporting); Project administration (equal); Supervision (lead); Validation (supporting); Writing‐original draft (supporting); Writing‐review & editing (supporting).

## Supporting information

Table S1

## Data Availability

The following will be uploaded to a publicly available Dryad repository upon acceptance: (i) RADseq data, used to ascertain SNPs for inclusion in the amplicon panel; (ii) GTseq data, used to genotype individuals; (iii) Individual genotypes with their sampled population, used to calculate per‐locus allele frequencies (given in the Table S1).
